# The Pharmacogenomics of Anti-Hypertensive Therapy

**DOI:** 10.3390/ph3061779

**Published:** 2010-06-01

**Authors:** Sandosh Padmanabhan, Laura Paul, Anna F. Dominczak

**Affiliations:** BHF Glasgow Cardiovascular Research Centre, 126 University Place, University of Glasgow, Glasgow G12 8TA, UK; E-Mails: 0506056P@student.gla.ac.uk (L.P.); a.dominiczak@clinmed.gla.ac.uk (A.F.D)

**Keywords:** hypertension, pharmacogenetics, pharmacogenomics, beta-blockers, ACE inhibitors, angiotensin receptor blockers, antihypertensive, calcium channel blockers, thiazide diuretics

## Abstract

Hypertension is a major public health problem, but measures to reduce blood pressure and thus cardiovascular risk are complicated by the high prevalence of treatment resistance, despite the availability of multiple drugs. Drug side-effects contribute considerably to suboptimal blood pressure control. Clinicians must often rely on empirical methods to match patients with effective drug treatment. Hypertension pharmacogenomics seeks to find genetic predictors of response to drugs that lower blood pressure and to translate this knowledge into clinical practice. In this review we summarise the current status of hypertension pharmacogenetics from monogenic hypertension to essential hypertension and discuss the issues that need to be considered in a hypertension pharmacogenomic study.

## 1. Hypertension—Scale of the Problem

Blood pressure (BP) is chiefly determined by cardiac output and total peripheral resistance. Hypertension is defined as a clinically significant increase in blood pressure, often considered to be DBP ≥ 90 mm Hg or SBP ≥ 140 mm Hg. This cut-off is somewhat arbitrary, as there is evidence of a continuous relationship between blood pressure and cardiovascular risk [1]. Hypertension using the dichotomous definition above is considered one of the most important preventable causes of premature death worldwide. Kearney and colleagues estimated the global burden of hypertension by searching the published literature from 1980 to 2002 [2]. They concluded that 26.4% of the global adult population had hypertension in 2000, and projected that this would increase to 29.2% by 2025. It was estimated that the total number of adults with hypertension would increase by over 60% during this time period from 972 million to 1.56 billion. The impact of the high prevalence of hypertension is substantial because of its large effects on mortality and morbidity. The Global Burden of Disease survey rated hypertension third on the list of factors responsible for the burden of disease during life, as measured by disability-adjusted life-years [3]. It has been proven beyond a doubt that a reduction in blood pressure is associated with decreased cardiovascular morbidity and mortality. A meta-analysis of 61 prospective, observational studies [1] covering 1 million adults and 12.7 million person-years, has demonstrated that for every 2 mm Hg decrease in mean systolic blood pressure, there is a 7% reduction in the risk of ischemic heart disease mortality, and a 10% reduction in the risk of stroke mortality. Lowering systolic blood pressure by 20 mm Hg reduced cardiovascular risk by half, irrespective of subject age [1].

## 2. Antihypertensive Therapy—Control and Resistance

A combination of drugs and life-style interventions are the cornerstones of hypertension treatment and cardiovascular risk reduction. While many safe and effective antihypertensive drugs exist, blood pressure is controlled in <50% of the patients. A recent analysis of National Health and Nutrition Examination Survey (NHANES), showed that only 53% of participants being treated for hypertension achieved a reduction in blood pressure below 140/90 mm Hg [4]. In a cross-sectional analysis of Framingham Heart Study participants, only 48% of participants on antihypertensive medication reached <140/90 mm Hg and less than 40% of elderly participants (>75 years of age) were at target blood pressure [5]. Among higher risk populations, particularly with the application of lower blood pressure goals in patients with diabetes mellitus or chronic kidney disease (CKD), the proportion of patients with poorly controlled BP is even higher. Uncontrolled hypertension can be secondary to poor adherence and/or an inadequate treatment regimen, as well as those with true treatment resistance. There is evidence that these poor rates of BP control are not explained by lack of treatment, as one study surveying 10,017 patients with hypertension estimated approximately 30% of treated hypertensives take one antihypertensive drug, 40% take two antihypertensives and 30% take three or more antihypertensives [6]. Prevalence of resistance to treatment has been estimated from large clinical outcome studies. In the Antihypertensive and Lipid-Lowering Treatment to Prevent Heart Attack Trial (ALLHAT) which included a large number of diverse participants (>33,000): 47% female, 35% African American, 19% Hispanic, and 36% with diabetes, 34% of participants remained uncontrolled on an average of two medications after approximately 5 years of follow-up [7]. Towards the end of the study, around 50% of the participants needed three or more drugs to achieve adequate BP reduction. This demonstrates the prevalence of treatment resistant hypertension, although the estimates are probably not true population estimates because of the inclusion criteria and the restrictions posed by the study treatment protocols. 

## 3. Variability in Antihypertensive Drug Response

The ability to identify patient characteristics associated with BP response to each drug class could increase control rates and improve on the current "trial-and-error" approach to selection of drug therapy for hypertension [8]. Some of these predictive factors include age, higher baseline BP, obesity, excessive dietary salt ingestion, chronic kidney disease, diabetes, left ventricular hypertrophy, black race, female sex, and measurements of the renin–angiotensin–aldosterone system (RAAS) [9,10]. Chapman *et al.* [11] showed that combined effects of all identifiable predictors (gender, age, ethnicity, body size, baseline BP, baseline levels of plasma renin activity (PRA), and urinary aldosterone excretion) accounted for 38 and 20% respectively of the interindividual variation in systolic and diastolic BP response to thiazide diuretics. In addition, genetic polymorphisms associated with both hypertension, *per se*, and BP response to various drugs are being reported with increasing frequency. 

## 4. Genetic and Non-Genetic Contribution to Drug Response

The promise of pharmacogenomics is that it may present a more effective way of identifying responders and non-responders to antihypertensive medication and those susceptible to adverse effects. Antihypertensive drugs lower blood pressure by acting on specific targets in the pathway of blood pressure regulation, although the mode of antihypertensive action of many of the available drugs is complex and multifactorial. Obvious candidate genes that influence drug responses are those that code for components of a system targeted by the drug or components of the counter-regulatory systems opposing the drug-induced fall in blood pressure. 

Ethnic differences in response to beta blockers and ACE inhibitors suggest different pathways are involved in hypertension causation in different ethnic groups. Indeed, antihypertensive drugs were the first cardiovascular therapies for which there was wide recognition of clinical differences in response based on ethnicity. Blacks generally respond more favourably to diuretics or calcium channel blockers, whereas whites tend to respond similarly to all the drug classes. In comparisons between groups, blacks respond slightly better than whites to diuretics and calcium channel blockers, whereas whites respond slightly better than blacks to ACE inhibitors and β-blockers [12]. If different pathways cause hypertension in different ethnic groups, then there is case for personalising therapy targeted towards the specific pathway based on ethnicity. However this is not straightforward, as there is considerable overlap between the two ethnic groups in terms of drug response [12].

This forms the basis of the theory underpinning the AB/CD algorithm [14,15]. Hypertension can be broadly classified as "high renin" or "low renin" based on the vasoconstriction-volume (renin/sodium) model of hypertension [16]. Renin-sodium profiling in patients with essential hypertension shows that the plasma renin activity (PRA) is increased in 15 percent, normal in 60 percent, and reduced in approximately 25 percent [16,17]. Low renin levels are found more frequently in blacks and in the elderly and in these individuals, the elevation in blood pressure is more likely to be salt-sensitive and the antihypertensive response may be greatest with a diuretic or calcium channel blocker [14,18]. It is proposed that initial treatment should be with one of two categories of antihypertensive drug: those that inhibit the renin–angiotensin system [angiotensin converting enzyme inhibitors (ACEIs; A) angiotensin receptor blockers (ARBs; A) or ß-adrenoceptor blockers (B)] and those that do not [calcium channel blockers (C) or thiazide diuretics (D)]. Individuals of white European ancestry and <55 years of age tend to have higher renin concentrations than those 55 of age or of African descent. A or B drugs are, therefore, generally preferred as initial blood pressure–lowering treatment in younger white patients than C or D drugs. However, C or D drugs are more effective first-line agents for those of African descent of any age or older white patients [19].

## 5. Monogenic Hypertension and Personalised Treatment

**Figure 1:**
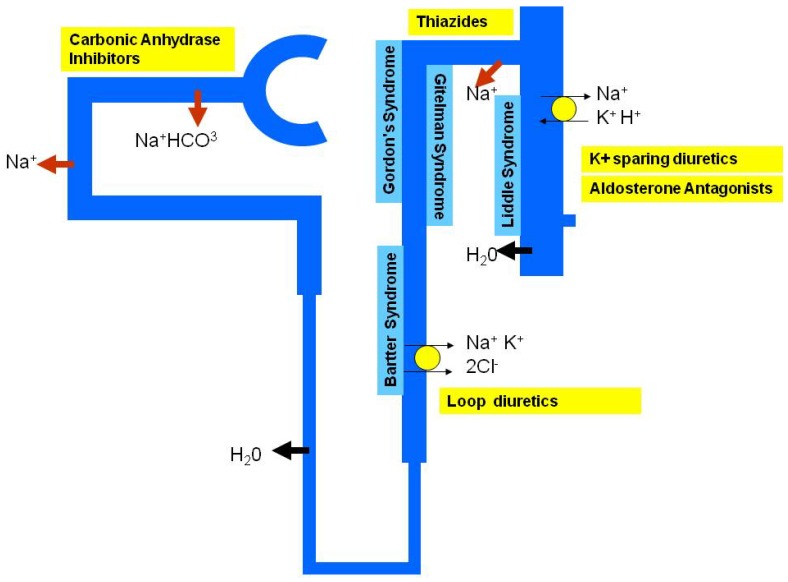
Schematic nephron showing sites of action of common diuretics and location of syndromes related to blood pressure regulation.

The search for the genetic determinants of antihypertensive drug response follows the same strategy as the studies of the genetics of complex traits. Most of the genetic studies of complex traits follow from successful gene mapping of rare monogenic forms of the condition. Rare monogenic forms of hypertension or hypotension have all been associated with genes involved in the renal tubular electrolyte transport functions [20] (Figure 1). There are about ten monogenic forms of hypertension and many of these are amenable to successful personalised treatment. We shall discuss some of the monogenic forms of hypertension and successful examples of targeted antihypertensive treatment based on genetic markers before moving on to essential or polygenic hypertension. 

Familial hyperaldosteronism type I (FH-I)—also known as glucocorticoid-remediable aldosteronism—was the first form of monogenic hypertension to be recognized as a single-gene hypertensive disorder [21]. The genetic defect is characterized by the presence of a chimeric gene on chromosome 8q consisting of the regulatory region of the 11-hydroxylase gene, *CYP11B1*, coupled with the structural region of the aldosterone synthase gene, *CYP11B2*. This results in aldosterone secretion in response to adrenocorticotrophic hormone (ACTH) in contrast to it usual agonists, angiotensin II and potassium. The specific treatment for hypertension in these individuals is low-dose glucocorticoids, amiloride, and spironolactone, which blocks binding of aldosterone to the mineralocorticoid receptor (MCR). 

A leucine for serine substitution at codon 810 in the hormone-binding domain of the MCR has been recently described, which causes the receptor to be constitutively active and alters its specificity such that the steroid hormones, which lack a 21-hydroxyl group and are normally antagonistic (including progesterone and cortisone), act as agonists [22]. Females with these mutations exhibit severe hypertension during pregnancy, exacerbated by the 100-fold rise in progesterone. Delivery of the foetus ameliorates the hypertension in pregnant females. No definitive therapeutic algorithm has been described for males or non-pregnant females, though spironolactone has been shown to aggravate the hypertension.

Liddle syndrome is an autosomal dominant disorder caused by hyperactivity of the amiloride-sensitive sodium channel (ENaC) of the principal cell of the cortical collecting tubule [23,24]. Mutations in the proline-rich regions of the beta and gamma subunits of the epithelial sodium channels result in the inability of these subunits to bind Nedd4 leading to constitutive expression of sodium channels at the apical surface of tubular cells. This causes increased rates of sodium reabsorption, volume expansion and hypertension. Liddle's syndrome can be regarded as the phenotypic extreme of low renin hypertension, with clear-cut salt-sensitive hypertension. Though in contrast to low renin essential hypertension where serum aldosterone levels are normal, in Liddle syndrome aldosterone levels are markedly suppressed. Treatment of Liddle syndrome with amiloride or triamterene lowers blood pressure and corrects the hypokalemia and acidosis. These agents effectively block the constitutively active ENaC in the collecting tubule. Spironolactone is not an appropriate treatment as the increased activity of the ENaC is not mediated by aldosterone.

Mutations of *WNK1* and *WNK4* (members of a family of serine–threonine kinases) have been shown to cause the rare familial autosomal dominant disease, Gordon's syndrome (pseudohypoaldosteronism type II) [25]. Wild-type *WNK1* and *WNK4* inhibit the thiazide-sensitive Na–Cl co-transporter in the distal tubule. Mutations of these proteins are associated with gain of function and increased co-transporter activity, excessive chloride and sodium reabsorption, and volume expansion. Treatment consists of either a low-salt diet or thiazide diuretics, aimed at decreasing chloride intake and blocking Na^+^–Cl^-^ co-transporter activity, respectively.

## 6. Essential Hypertension and Personalised Treatment

There is considerable evidence supporting the presence of a modest genetic contribution to the common essential hypertension which poses the significant public health problem as described above. Studies of blood pressure in twins and familial aggregation of blood pressure indicate a heritable component to non-Mendelian hypertension. Hypertension is about twice as common in individuals who have one or two hypertensive parents, and blood pressure is more closely correlated in identical (monozygotic) than non-identical (dizygotic) twins [26,27]. In the Montreal Adoption Study investigators compared blood pressure correlation between biological sibling pairs and adoptive sibling pairs (as well as parent-child correlations). Systolic BP (SBP) correlation coefficients were 0.38 and 0.16 for biological and adopted siblings respectively, and Diastolic BP (DBP) coefficients were 0.53 compared with 0.29 respectively [28]. Genome-wide linkage approach with microsatellites has shown evidence for existence of several chromosomal regions that are linked to BP or essential hypertension on almost all chromosomes [29,30]. However linkage mapping has been difficult because of the polygenic nature of hypertension, possibly involving multiple pleiotropic variants of low penetrance, epistasis, ethnic diversity of human populations, phenotypic heterogeneity and the inability to control for environmental factors. In addition, linkage analysis has poor power for detecting common alleles that have low penetrance. All these factors hinder the replication of results and decrease the likelihood of success of linkage studies in general. The Medical Research Council funded British Genetics of Hypertension (MRC BRIGHT) study, [31] demonstrated a successful attempt at reducing heterogeneity by using antihypertensive drug response to partition different pathways of hypertension [32]. In the BRIGHT population, hypertensive sib-pairs who were non-responsive to ACE inhibitors, ARBs or beta-blockers showed significant linkage on chromosome 2p (LOD = 4.84 at 90.68 Kosambi cM). This susceptibility locus co-localises to a region found in African-American hypertensive patients in the Family Blood Pressure Program, who showed evidence of linkage with hypertension status at 93 cM with a LOD score of 2.84 [33]. Thus the chromosomal 2p locus independently identified in different populations may contain a gene or genes for the salt-sensitive form of hypertension which is common among Africans, and the same mechanism may be operative in a subset of white European hypertensive patients identified by unresponsiveness to beta blockers and ACE inhibitors. However, this study needs to be replicated and the underlying causative gene identified before translation.

There are numerous small candidate gene association studies that have tried to determine if common polymorphisms in genes can predict response to antihypertensive agents. The candidate gene association study approach involves the selection of candidate genes based on a mechanistic understanding of the roles of the encoded proteins in blood pressure regulation, or drug target. However this will, at best, identify only a fraction of genetic risk loci even if the pathway is relatively well understood. Candidate genes studied fall into the following categories—the Renin-Angiotensin-Aldosterone-System, adrenergic system and genes involved in sodium transport in the kidneys. None of the candidate gene studies have so far shown reproducible associations. Some of the limitations of the candidate gene approach are as follows: first, the choice of candidate genes may be inappropriate. Second, the causative genes might be either upstream of the points of action or in the downstream signalling pathways of the selected candidates. Third, the SNPs selected for association studies may provide incomplete coverage of all the variants within the genes studied. Fourth, most studies are underpowered and problems due to population stratification, phenotypic and locus heterogeneity. Finally, candidate gene studies rely on prior hypotheses about disease mechanisms, so that discovery of genetic variants in previously unknown pathways is precluded [34]. The most convincing candidate gene study is the *ACE* I/D polymorphism in the pharmacogenetic substudy of ALLHAT, called GenHAT. This study tested the association of various outcomes in ALLHAT with the I/D polymorphism in 37,939 patients. No association was found between this polymorphism and BP lowering (with lisinopril or any other study drugs) nor with any of the study outcomes, either when considered in combination or stratified by drug therapy [35]. Similarly in the large INVEST study, no genetic association was found between *AGTR1* 1166A→C genotype and BP response [36]. In an association study of 585 subjects, between diuretic response and genes in renal sodium transport systems, polymorphisms in *WNK1*, *ADRB2*, and the epithelial sodium channel-subunit gene (*SCNN1G*) predicted interindividual differences in antihypertensive responses to hydrochlorothiazide [37]. The association between BP lowering with beta-blockers and genetic polymorphisms in the beta1-adrenergic receptor gene (*ADRB1*)- Ser49Gly and Arg389Gly, suggest greater BP lowering in the Arg389Arg individuals. The commonly studied is the Arg389Gly polymorphism, for which many, but not all, studies show a significant association with antihypertensive response to beta-blockers and two independent studies have suggested an association between treatment related hypertensive outcomes and ADRB1 SNPs [38,39,40]. There is some evidence that the Ser49Gly polymorphism alone does not importantly influence BP response, but when considered in combination with the Arg389Gly polymorphism, it may be more informative than Arg389Gly alone [41,42]. The Gly460Trp polymorphism of the alpha Adducin (*ADD1*) gene is another extensively studied candidate gene and one study showed differential outcomes (myocardial infarction or stroke) with thiazide treatment based on the Gly460Trp genotype [43]. But, subsequent outcomes-based analyses from controlled clinical trials did not replicate this finding [44,45]. However, *ADD1* gene remains an interesting candidate and recently *ADD1* considered together with *NEDD4L* revealed a significant association with BP response to a thiazide, while neither gene alone showed such an association [46]. In addition to epistasis, interactions among the three adducin subunits as well as tissue-specific splicing isoforms need to be considered for a more thorough dissection of adducin and its role in antihypertensive response [47]. This supports the polygenic nature of antihypertensive response and highlights the challenges to dissect this trait. Another potential candidate gene is *KCNMB1*, for which SNPs have been associated with BP response in three independent populations, and treatment-related outcomes in one [48,49].

Finally the first hypertension pharmacogenomics genome-wide association study (GWAS) showed promise by discovering and validating a region on chromosome 12 that was associated with the response to hydrochlorothiazide [50]. With decreasing cost of genotyping and successful heritage of validated disease associations, GWAS will be a strategy of choice in the near future for pharmacogenomic studies. 

## 7. Designing a Pharmacogenetic Study of Antihypertensive Treatment

There are important design issues to be considered in antihypertensive pharmacogenomics studies. Kurland *et al.* [51] have suggested the ideal study should follow certain general principles: studies should be prospective, include previously untreated hypertensive individuals, treat with one drug at a time from each drug class on a random, rotational basis and there should be a placebo controlled arm. All such studies should be followed by independent replication. However, this is not practical either in terms of logistics or ethics (placebo studies in hypertension are not acceptable). As alluded to above, adequate sample size and power are key to the success of any study, and this applies to pharmacogenetic studies equally. Recently the identification of a SNP in the *SLCO1B1* gene, encoding the organic anion-transporting polypeptide *OATP1B1*, as a susceptibility factor for statin-induced myopathy involved a genome-wide association analysis of 85 individuals with definite or incipient statin myopathy (and 90 controls) from a trial involving over 12,000 subjects [52]. Here, though the final sample sizes are very small, this should in no way lead to underestimating the large scale effort needed to identify the few subjects who suffer extreme adverse effects. The key to success here is phenotypic specificity and this is an aspect that is very relevant to hypertension pharmacogenetic studies. For measures of drug response, the unimodal population response to drugs suggests that pharmacokinetics and dynamics are also polygenic traits that can be dissected as other complex disease traits.

The study design that is most likely to yield validated results would be GWAS but one should keep in mind the limitations of GWAS. All SNP chips used for GWAS genotype common variants and with the high coverage in newer chips which can genotype a million SNPs per sample along with imputation using data from the 1000 genome project (http://www.1000genomes.org), one can be confident of interrogating almost the entire genome in terms of common variants. However, these experiments are blind to rare variants and structural variants. Though GWAS has identified numerous validated SNPs for most common diseases and traits, these explain only a tiny proportion of the heritability of these traits suggesting that rare variants and structural variants may explain the remaining missing heritabilities [53]. However, these require sequencing and next generation sequencing technologies are becoming more accessible because of innovations in technology and decreasing costs. These studies by analysing hundreds of thousands of variants, need appropriate methods to address multiple testing issues. Currently the threshold used id p < 5 × 1 × 10^-8^, however the gold standard of any study is multiple replications in independent studies followed by functional validation [54,55].

Interpretation of the results of association studies is dependent on a narrow definition of the phenotype. For blood pressure this is difficult, in view of the variability of blood pressure, and a significant association result for blood pressure traits may in fact be an association between the factors that introduce variability in the measures [56]. A multitude of external factors can affect blood pressure measurements at each visit. These factors include uncalibrated sphygmomanometers, zero-digit bias, the effects of taking blood tests, the influence of the rest period, cuff size *etc.* [57]. While the differences in measurement may appear small, there is evidence of significant misclassification, as most errors result in overestimates of blood pressure. There are suggestions that using a more robust phenotype like ambulatory blood pressure would reduce errors. One reason is that mean ambulatory blood pressure is much more reproducible than office blood pressure. This is because it is a mean of multiple measurements and is less prone to environmental noise [58]. The alerting reaction, for example, exists for office but not for ambulatory mean blood pressure [59]. 

Clinical trials which have shown blood pressure reduction on placebo treatments suggest that much of the apparent fall in blood pressure seen in clinical practice is regression to the mean [60]. In other words, the average blood pressure is overestimated before treatment and all this is due to large day-to-day, within-individual blood pressure variability. The coefficient of variation is at least 8% and may be very much higher. Some studies have observed pharmacogenetic associations only in certain population substrata. While significant reductions in power can occur when cohorts are divided for subgroup analysis, there are specific problems with sexual dimorphism in the blood pressure phenotype. Longitudinal studies have shown a gender difference in blood pressure in early adolescence and a link with onset of puberty and later blood pressure supporting a hormonal contribution [61,62]. This sexual-dimorphism has been shown to affect the results of genetic studies – both linkage and association [56,63].

## 8. Conclusions

Genetic predictive testing for antihypertensive dose adjustment of avoidance of adverse events require large scale studies taking into account the complexities of the blood pressure phenotype. Narrowing the phenotype by focussing on pathways will make this genetically tractable and lead to successful clinical translation. The discovery of new drug targets is no less important and the large scale efforts in whole genome mapping of hypertension and blood pressure traits will be key in this aspect.
